# Mentalizing partially mediates the association between attachment insecurity and global stress in preservice teachers

**DOI:** 10.3389/fpsyg.2023.1204666

**Published:** 2023-08-21

**Authors:** Nicola-Hans Schwarzer, Lars Dietrich, Stephan Gingelmaier, Tobias Nolte, Tijs Bolz, Peter Fonagy

**Affiliations:** ^1^Heidelberg University of Education, Heidelberg, Germany; ^2^Humboldt University of Berlin, Berlin, Baden-Wurttemberg, Germany; ^3^Ludwigsburg University, Ludwigsburg, Baden-Württemberg, Germany; ^4^Anna Freud National Centre for Children and Families, London, United Kingdom; ^5^University of Oldenburg, Oldenburg, Lower Saxony, Germany; ^6^University College London, London, England, United Kingdom

**Keywords:** attachment, stress, mentalizing, teacher, preservice teacher

## Abstract

**Objective:**

Considering the fact that the teaching profession is a highly stressful occupation and that teachers’ ineffective coping strategies contribute to higher levels of stress, the objective of the present study was to investigate whether insecure attachment is related to global stress experiences in preservice student teachers. Furthermore, it was examined whether this link is mediated by the preservice teachers’ mentalizing—the capacity to perceive and consider one’s own and others’ behavior based on intentional mental states.

**Methods:**

Data of this cross-sectional study came from 202 preservice student teachers using self-report instruments (attachment: Expectation in Close Relationships; mentalizing: Reflective Functioning Questionnaire; stress: Trier Inventory of Chronic Stress). The hypotheses were tested using structural equation modelling.

**Results:**

Anxious attachment was positively related to stress and impairments in mentalizing. In addition, the link between attachment-related anxiety and stress was partially mediated by mentalizing. Avoidant attachment was not associated with stress or mentalizing.

**Discussion:**

Results indicate that anxious attachment is associated with higher perceived stress in preservice student teachers. Consequently, attachment-related anxiety may be a risk factor which, in turn, may foster perceptions of higher global stress experiences.

**Conclusion:**

Additional research needs to focus on exploring the link between attachment insecurity and global stress experiences among preservice student teachers. An examination of preservice student teachers’ own attachment experiences proves to be useful, for example in the context of mentalization-based supervision or reflective practice.

## Introduction

The Transactional Stress Model (Lazarus and Folkman, 1984, 1987) conceptualizes stress as an interaction between a person and their environment, in which the person experiences challenges that exceed their resources or threaten their well-being. This imbalance is accompanied by a hypervigilant psychophysiological state of arousal leading to stress-related symptoms of emotional exhaustion, which can lead to lasting physical and mental health impairments (Klusmann and Waschke, 2018). Teachers are an occupational group that experiences particularly high levels of stress and pronounced stress-related symptoms of emotional and physical exhaustion (e.g., Hasselhorn and Nübling, 2004; Lohmann-Haislah, 2012; Nübling et al., 2012). This burden is associated with limitations in subjective well-being (Meng and D’Arcy, 2015) and has been linked to negative teaching behaviors in the classroom (e.g., Klusmann et al., 2006, 2016). For example, Shen et al. (2015) found that teachers’ stress related emotional exhaustion at the beginning of the semester was a negative predictor of students’ motivation at the end of the semester.

Rothland (2013) proposed that contextual factors such as having a split workplace (school and home), unregulated working hours, coercion-based cooperation with students, or low opportunities for promotion can lead to high levels of stress experience among teachers. Research further suggests that classroom disruptions and student discipline problems are additional factors that predict higher levels of stress in teachers (e.g., Chang, 2009; Tsouloupas et al., 2010). However, Hillert et al. (2013) suggested that these challenges do not necessarily lead to high stress experiences for all teachers on an individual level. In line with this, Klusmann et al. (2008) found that contextual factors such as school size, the size of taught courses, the school district, the socioeconomic status of students, and their cognitive abilities are only slightly associated with stress-related symptoms of exhaustion among teachers. Instead, research suggests that negative personal characteristics, such as maladaptive patterns of self-regulatory abilities (e.g., Schaarschmidt et al., 1999; Schwarzer et al., 2022a) are more strongly associated with higher stress experiences. For instance, Lehr et al. (2008) found that teachers with negative patterns of coping strategies characterized by the tendency to resign report strong impairments in well-being, extensive emotional exhaustion, and higher depressive symptoms with overall large effect sizes. Notably, these results were independent of the average number of teachers’ working hours (part-time; full-time). These findings are of great importance: Apparently, high levels of stress-related exhaustion are not caused by the amount of time teachers spend in the classroom, but are instead linked to personal characteristics that are risk factors among prospective teachers for experiencing higher levels of stress even before they enter the profession.

As a consequence to these findings, Klusmann and Waschke (2018) have suggested that empirical studies need to pay greater attention to teachers’ personal characteristics and evaluate their impact on the development of global stress experiences at an early stage of teacher education. The university training phase of teachers reflects a promising window of prevention, as contextual and school-characteristic features seem to play a less important role in the development of stress than personal factors (Hillert et al., 2013). Therefore, it is important to focus on these personal features in the curriculum within the close cooperation between student teachers and lecturers at university prior to their entering teaching service in the field. This approach could help identifying individual differences and the underlying psychological mechanisms in early stages of teacher education and training, in order to prevent a more negative professional development of teacher candidates later on.

Attachment theory, although developed 60 years ago, continues to be considered an influential developmental theory in the behavioral and social sciences (Thompson et al., 2022). This theory, first proposed by John Bowlby (1944) suggests that humans have an innate drive to form close emotional relationships with attachment figures in order to create and maintain physical and psychological proximity (Bowlby, 1988). According to Bowlby (1969, 1988), when a child is exposed to threatening stimuli, the child’s attachment system is activated, leading to attachment behavior such as crying, calling, or seeking out the attachment figure. This behavior then triggers caring behavior from the attachment figure (Bowlby, 1969, 1988) creating a co-regulatory system that helps the child cope with stressful experiences (Bowlby, 1988).

The long-term effects of attachment relationships on the development of cognitive-affective representations, referred to as Internal Working Models [IWMs] have been studied extensively in recent decades (Bretherton, 1990; Bretherton and Munholland, 2018). IWMs are thought to be relatively stable over time (e.g., Fraley and Shaver, 2000; Mikulincer and Shaver, 2016), and shape behavior in challenging situations of allostatic load (Bowlby, 1988; Bretherton and Munholland, 2018).

Secure attachment representations, developed by the child on the basis of sensitive caring behavior by the attachment figure (Ainsworth et al., 1978), are considered an important factor positively influencing psychosocial development and mental health (Groh et al., 2014). In such cases, the attachment figure serves as a “safe haven” by providing the child with reliable and co-regulatory support in stressful or threatening situations (Bowlby, 1988). This leads to the development of attachment representations in which the child is represented as worthy of protection and self-efficacy, and which shape beliefs that even stressful situations are modifiable (Bretherton and Munholland, 2018). On the other hand, insecure attachment representations—caused by caregiving behavior that was dismissive or barely predictable—reflect a risk factor that has been consistently linked to a whole series of negative outcomes such as mental health impairments (Zhang et al., 2022), internalizing symptoms (Groh et al., 2012), externalizing symptoms (Fearon et al., 2010), or impairments in emotional (Groh et al., 2017) and social competencies (Groh et al., 2014).

Ainsworth et al.’s (1978) extension of Bowlby’s theory of attachment distinguished two forms of attachment insecurity: insecure-ambivalent and insecure-avoidant (Ainsworth et al., 1978). Insecure-ambivalent attachment is characterized by high levels of anxiety due to highly inconsistent and difficult to predict experiences with primary caregivers during stressful situations (Bretherton and Munholland, 2018). This leads to aversive experiences being highly stress-inducing, with little coping capacity available, and resulting in strong feelings of helplessness (Dykas and Cassidy, 2011). Insecure-avoidant attachment is argued to be rooted in experiences with primary caregivers who have been consistently rejecting and unavailable in stressful situations (Bretherton and Munholland, 2018). In these cases, attachment-related needs and whishes triggered in distressing situations are suppressed in a defensive manner as a way of self-protection despite significant psychophysiological arousal (Mikulincer et al., 2003; Dykas and Cassidy, 2011).

Mentalizing, or the ability to perceive and consider one’s own and others’ behavior as based on intentional mental states, is closely linked to attachment theory (Fonagy et al., 1991; Fonagy and Allison, 2014). It is conceptualized as a developmental achievement associated with the increasing awareness of the importance of mental states in organizing behavior (Fonagy et al., 2002; Luyten et al., 2020). Secure attachment relationships are thought to provide an adaptive learning environment in which children can develop effective mentalizing skills as a result of having their feelings reflected back sensitively by their attachment figures (Fonagy et al., 2002). Conversely, insecure attachment relationships can compromise the development of children’s mentalizing capacities due to the poorly attuned interaction experiences between the children and the attachment figures (Luyten et al., 2017).

Mentalizing has become an increasingly important aspect of mental health (Katznelson, 2014). Research has shown that mentalizing skills are impaired in patients with depression (Fischer-Kern et al., 2022), borderline personality disorder (Németh et al., 2018), pathological anxiety (Chevalier et al., 2023), or antisocial personality disorder (Newbury-Helps et al., 2017) compared to healthy controls. Further, psychotherapeutic interventions have been found to promote mentalizing (Levy et al., 2006; Fischer-Kern et al., 2015; Storebø et al., 2020), which in turn is associated with a decrease in symptom severity (Rossouw and Fonagy, 2012; De Meulemeester et al., 2018).

Recently, the focus on mentalizing as a clinically important factor in psychopathology has been extended to an understanding of mentalizing as a coping strategy buffering the negative impact of aversive experiences (Fonagy et al., 2017; Luyten et al., 2020). It has been argued that mentalizing protects against the effects of stressful experiences and enables a more resilient adaptation to, and processing of, stress (Fonagy et al., 2017; Holmes, 2017). This hypothesis assumes that mentalizing enables individuals to manage, process, and give meaning to their own and others’ behaviors, which helps them to maintain a robust comprehension of social interactions (Fonagy et al., 1994, 2002). Hence, mentalizing as an intrapsychic coping strategy is believed to reduce experiences of distrust, confusion and loss of control when confronted with emotionally challenging situations, and to foster a stable sense of self-coherence (Fonagy et al., 2019, 2021). Consistent with these assumptions, empirical data suggest that mentalizing might serve as a mediating state of mind reducing the impact of adversity in clinical (e.g. Bizzi et al., 2020; Huang et al., 2020) and non-clinical samples (e.g. Brugnera et al., 2021).

### The current study

Attachment theory (Bowlby, 1969, 1988) and the concept of IWMs (Bretherton, 1990) suggest that associations between attachment insecurity and stress experiences are likely to occur throughout the lifespan. Mikulincer et al. (2003) proposed that IWMs serve as a mental mechanism that allows for adaption and self-regulation even in adulthood (Simpson and Rholes, 2017). Mikulincer and Shaver (2016) further suggested that insecure attachment representations may lead to more intense experiences of stress through three different aspects: First, attachment insecurity may foster the use of maladaptive emotion regulation strategies to cope with external stressors. Second, it may lead to negative self-representations, thus lowering self-esteem in the face of external stressors. Third, attachment insecurity is linked to chronic interpersonal problems, which can lead to a subjectively perceived imbalance between stressors and available coping resources, resulting in higher stress experiences (Lazarus and Folkman, 1984, 1987).

The existing research on the link between insecure attachment and stress experiences provides evidence from both self-reported data (e.g., Karreman and Vingerhoets, 2012; Li and Zheng, 2014; Thompson et al., 2022) and psychophysiological data (e.g., Ehrenthal et al., 2010; Pierrehumbert et al., 2012). The findings suggest that attachment-related avoidance and attachment-related anxiety are associated with heightened stress experiences. Furthermore, evidence from an experimental setting (Ehrenthal et al., 2018) has demonstrated that attachment insecurity in adults can act as a moderator between adverse experiences and physiological stress response, suggesting that attachment representations may have a life-long stress-regulating effect (Tironi et al., 2021).

Recent research has demonstrated that mentalizing may play an important role in mitigating the negative association between attachment insecurity and global stress experiences. With the emergence of a clinical focus on mentalizing as a health-promoting capacity (Luyten et al., 2020), this capacity is conceptualized as a potentially mediating mechanism in the mental processing of aversive experiences by enhancing coherent self-awareness in high stress settings and thus, maintaining feelings of agency (Fonagy et al., 2017). Preliminary empirical data have provided support for this hypothesis, demonstrating the effectiveness of mentalizing in reducing the use of dysfunctional coping behaviors (e.g., Chiesa and Fonagy, 2014; Taubner et al., 2016; Schwarzer et al., 2022b) and compensating for the negative impact of insecure attachment on occupational effectiveness (Cologon et al., 2017).

The current study investigated the associations between attachment insecurity and global stress experience in prospective teachers, which have not yet been studied. It was further hypothesized that mentalizing capacity would mediate the positive associations between attachment-related anxiety or attachment-related avoidance and global stress experiences. Although similar associations have been studied in clinical (e.g., Badoud et al., 2018; Borelli et al., 2018) and non-clinical populations (Bordoagni et al., 2021; Brugnera et al., 2021) the effects of attachment insecurity or mentalizing capacity on global stress experiences in an educational context are still unknown. At least some research points into the health-preserving function of effective mentalizing in educators (Schwarzer, 2019; Levante et al., 2023; Safiye et al., 2023; Schwarzer et al., 2023). Moreover, evidence suggests that avoidant attachment representations are overrepresented in educational samples compared to the general population (Hedervari-Heller and Antunes, 2017); however, the associations between insecure attachment representations and stress parameters in educators is yet to be documented (Schröder et al., 2022), although attachment theory (Bowlby, 1988) and the lifelong regulatory function of attachment representations (Mikulincer and Shaver, 2016) suggest this may be the case.

**Figure 1:**
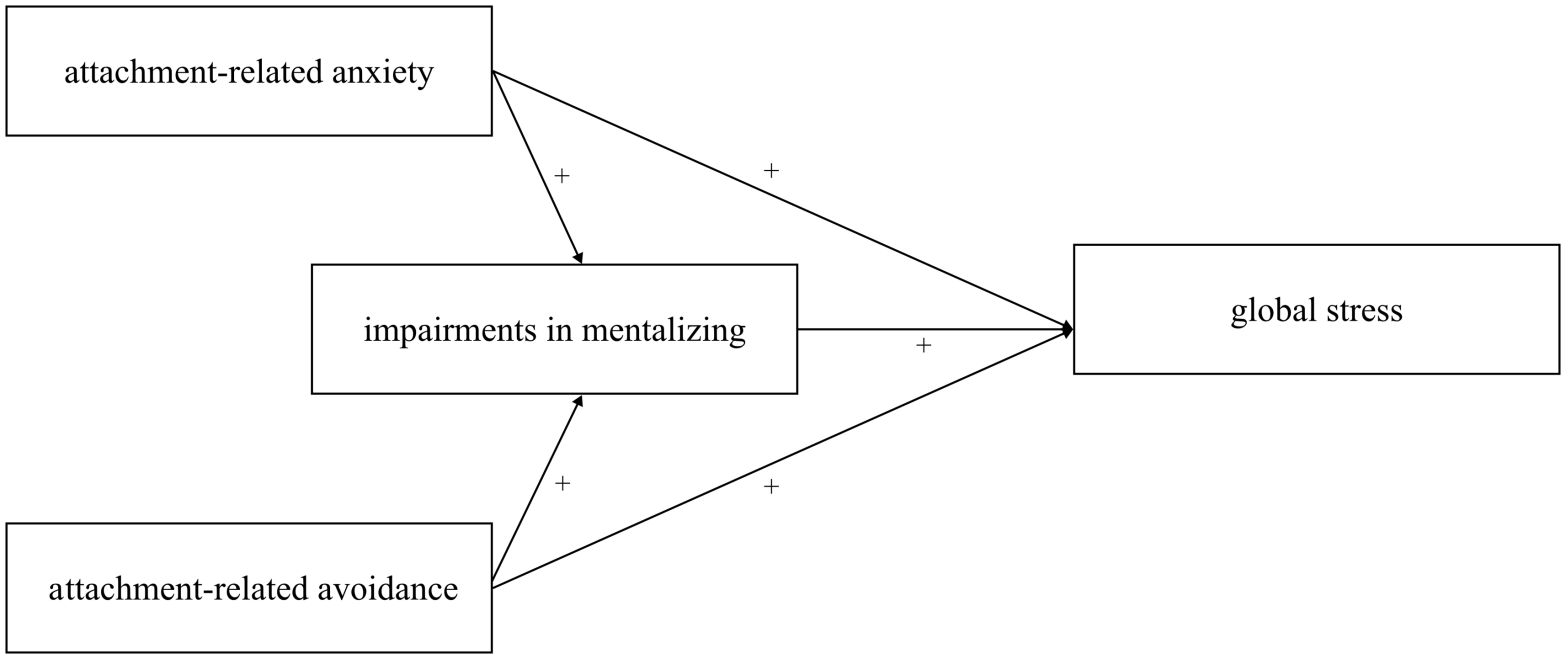
Hypothesized model.

The purpose of this study was to examine the relationship between attachment-related anxiety, attachment-related avoidance, mentalizing, and global stress in preservice teachers. Specifically, Hypothesis 1 postulated that attachment-related anxiety would be positively associated with global stress experiences in preservice teachers. Hypothesis 2 suggested that attachment-related avoidance would be positively linked to global stress experiences in preservice teachers. Hypothesis 3 proposed that mentalizing would mediate the association between both forms of insecure attachment and global stress in preservice teachers (see Figure 1).

## Materials and methods

### Participants and procedure

This cross-sectional study examined the attachment representations, global stress experiences and mentalizing capacities of 202 preservice student teachers (171 female; 31 male). Data was collected from October 2021 to February 2022. Due to Covid pandemic-related restrictions, the study used the online survey platform UniPark for data collection. The average age of the sample was 25.61 (*SD* = 5.64; *min* = 19; *max* = 53) years with no significant age differences between female and male students (*χ^2^* = 36.93; *p* = 0.18). Further, data analysis revealed no differences between the study variables based on native language or gender (see: Electronical supplement). All subjects provided written informed consent to participate in the study, which was voluntary and could be withdrawn at any time. The research program was approved by the Ethics Committee of the Ludwigsburg University of Education (III-Sopead_NiSc-0009).

### Measures

#### Attachment insecurity

In this study, the short form of the German version of the Experiences in Close Relationships—Revised Questionnaire (ECR-RD8) was used to assess attachment insecurity, which was recently evaluated with a large German sample (Ehrenthal et al., 2021). ECR-RD8 is a self-report measure consisting of eight statements to be answered on a seven-point Likert scale from 1 (strongly disagree) to 7 (strongly agree). The measure has two subscales: attachment-related anxiety (e.g., “I rarely worry about my partner leaving me”) and attachment-related avoidance (e.g., “It’s not difficult for me to get close to my partner” [reverse coded]) with high scores on both scales indicating stronger expressions of attachment insecurity. Ehrenthal et al. (2021) reported good psychometric characteristics for ECR-RD8, which were confirmed in this study (ERC_ANX: *α* = 0.87; *ω* = 0.87; ECR_AVOI: *α* = 0.73; *ω* = 0.73). Kolmogorov–Smirnov-tests indicated that both subscales were not normally distributed (both *p* < 0.001). However, the skewness and kurtosis values for both subscales were within the acceptable range suggested by West et al. (1995) (skewness ≤ |2|; kurtosis ≤ |7|), indicating a sufficient level of normality.

#### Mentalizing

The current study used a short version of the Reflective Functioning Questionnaire (RFQ; Fonagy et al., 2016) to assess mentalizing, relying on the individual self-reported tendencies to consider mental states as relevant in the understanding of one’s own behavior and the behavior of other people (e.g., “I do not always know why I do what I do”). RFQ is a reliable and valid instrument that is suitable for use in larger samples and comprises eight statements that the subject is asked to rate on a seven-point Likert scale from 1 (completely disagree) to 7 (completely agree). Building on a large German community sample, Spitzer et al. (2021) recommend a one-dimensional, six-item scale that exclusively assesses uncertainty in using mental states as reliable information, which was recently replicated by Wozniak-Prus et al. (2022). Therefore, the items 4 and 7 were excluded. Higher values relate to higher uncertainty in considering mental states as relevant, indicating stronger impairments in mentalizing. In the current study the internal consistency was acceptable (*α* = 0.76; *ω* = 0.78). A Kolomogorov–Smirnov-Test indicated normality (Kolomogorov–Smirnov-test: *p* = 0.149).

#### Stress experiences

Following the Transactional Stress Model (Lazarus and Folkman, 1984, 1987), this study assessed global stress experiences with the screening scale of the Trier Inventory of Chronic Stress (TICS; Schulz et al., 2004), a reliable and valid instrument for assessing transactional stress experiences with good internal consistency (*α* = 0.91; *ω* = 0.91). Participants rated 12 statements on a five-point Likert scale (0 = never to 4 = very often; e.g., “How often have you experienced the following in the last 3 months: Fears of not being able to perform my assignments”). The Kolomogorov–Smirnov-test did not reject the null-hypothesis of normality (Kolomogorov–Smirnov-Test: *p* = 0.36), suggesting that a normal spread of the scores can be assumed. High scores on the TICS indicated higher levels of stress. The instrument is considered economical for studying large samples (Schulz et al., 2004).

### Statistical analyses

A power analysis was conducted using g-power (Faul et al., 2007) to determine the necessary sample size. Based on the results reported by Brugnera et al. (2021), a medium effect size of *f^2^* = 0.15 was used. With six predictors, a power of *β* = 0.95 and *α* of 0.05 a sample size of *N* = 146 was needed. Data analyses were performed using AMOS 29 and SPSS 29. With 0.29% of the data missing (Little-test: *χ*^2^ = 1511.15; *p* = 0.636), imputation was done using the expectation–maximization algorithm (Tabachnick and Fidell, 2012). No multivariate outliers were observed (Mahalanobis distance; Tabachnick and Fidell, 2012). Age, native language (1 = German; 2 = other), and gender (1 = female; 2 = male; 3 = divers) were entered as covariates in all analyses. The associations between all variables were explored using robust correlation coefficients (Spearman) and partial correlations controlled for gender, native language, and age in a first step. Given the moderate violation of multivariate normality as evidenced by Mardia’s normalized multivariate kurtosis, the bootstrapping maximum likelihood estimator with 10,000 bootstrap samples was used to calculate robust standard errors, which is recommended in structural equation modeling (SEM) under non-normal data conditions (Nevitt and Hancock, 2001; Hayes, 2009). To test the hypothesized model, SEM (maximum likelihood estimator) was used with “attachment-related anxiety” and “attachment-related avoidance” as the exogenous variables and “global stress experiences” as the dependent variable. “Impairments in mentalizing” were entered as a mediating variable. All variables—attachment-related anxiety, attachment-related avoidance, impairments in mentalizing, and current stress experiences – were modeled as latent variables, which were tested using confirmatory factor analyses (CFA) in a first step (Anderson and Gerbing, 1988). In a second step, SEM was conducted to test the hypothesized model (Anderson and Gerbing, 1988). To evaluate the models, the following fit indices were used (Hu and Bentler, 1999): (1) the *χ*^2^ statistic, (2) the root mean square error of approximation (RMSEA) with its 90% confidence interval (CI), (3) the comparative fit index (CFI), and (4) the Standardized RMR (SRMR; excellent fit: non-significant *χ^2^* statistic, RMSEA ≤ 0.06, CFI ≥ 0.95; SRMR ≤ 0.06); acceptable fit: non-significant *χ*^2^ statistic, RMSEA ≤ 0.08, CFI ≥ 0.90; SRMR ≤ 0.08). Furthermore, due to the large sample size (> 200) a significant *χ*^2^ statistic was expected. Direct and mediation effects were examined using the bootstrap CI method with 10,000 bootstrap samples, and 95% CIs were analyzed (Hayes, 2009). Age, gender and native language were included as controls in all further analyses.

## Results

### Preliminary data analyses

Table 1 provides descriptive statistics and intercorrelations between attachment-related anxiety, attachment-related avoidance, impairments in mentalizing, and global stress experiences in the studied sample. Moderate positive associations between attachment-related anxiety, mentalizing impairments, and stress experience were found, as measured by Spearman correlation coefficients and partial correlation coefficients controlling for gender identity, native language, and age. Attachment-related avoidance, impairments in mentalizing, and global stress experience were also positively associated, though the overall correlations were lower.

**Table 1:** Descriptives and intercorrelations (*n* = 202).

	1	2	3	4
1 ECR_ANX	–	0.480^***^	0.299^***^	0.430^***^
2 ECR_AVOI	0.467^***^	–	0.265^***^	0.191^***^
3 RFQ	0.373^***^	0.320^***^	–	0.432^***^
4 TICS	0.485^***^	0.240^**^	0.475^***^	–
Mean	9.73	8.70	18.73	34.55
SD	5.68	4.31	5.97	8.38
*α*	0.87	0.73	0.73	0.91
*ω*	0.87	0.73	0.73	0.91
Skewness	1.14	1.61	0.45	0.16
Kurtosis	0.49	3.91	0.03	−0.32

*N* = 202. ECR = Expectations in Close Relationships—Revised Questionnaire. ECR_ANX refers to the attachment related anxiety subscale of the ECR. ECR_AVOI refers to the attachment related avoidance subscale of the ECR. RFQ = Reflective Functioning Questionnaire. TICS = Trier Inventory of chronical stress. All correlation coefficients above the diagonal were estimated using a distribution-free measure (Spearman). All correlation coefficients below the diagonal were controlled for the influence of age, native language, and gender. ^***^p < 0.001, ^**^p < 0.01, ^*^p < 0.05.

Before SEM was used, each measurement model was tested in a first step. A general factor of attachment-related anxiety was modeled, using all four items of the ECR attachment-related anxiety subscale, revealing acceptable fit (*χ*^2^(1, *n* = 202) = 2.356, *p* = 0.13, RMSEA = 0.08 [0.000–0.22], CFI = 1.00, SRMR = 0.01). Attachment-related avoidance was derived from all four items of the ECR attachment-related avoidance subscale, with acceptable fit (*χ*^2^(2, *n* = 202) = 1.35, *p* = 0.51, RMSEA = 0.00 [0.00–0.13], CFI = 1.00, SRMR = 0.02). A general factor of impairments in mentalizing was derived from the RFQ using the six items recommended by Spitzer et al. (2021), revealing acceptable fit (*χ*^2^(8, *n* = 202) = 7.70, *p* = 0.46, RMSEA = 0.00 [0.00–0.08], CFI = 1.00, SRMR = 0.03). Global stress experiences were estimated through all 12 items of the TICS. The latent variable showed an adequate fit (*χ*^2^(46, *n* = 202) = 77.66, *p* = 0.002, RMSEA = 0.06 [0.05–0.08], CFI = 0.98, SRMR = 0 0.04). All of the loadings of the manifest variables on the latent factors were also found to be statistically significant (*p* < 0.001) with factor loadings higher than 0.40.

### Structural equation modeling

**Figure 2:**
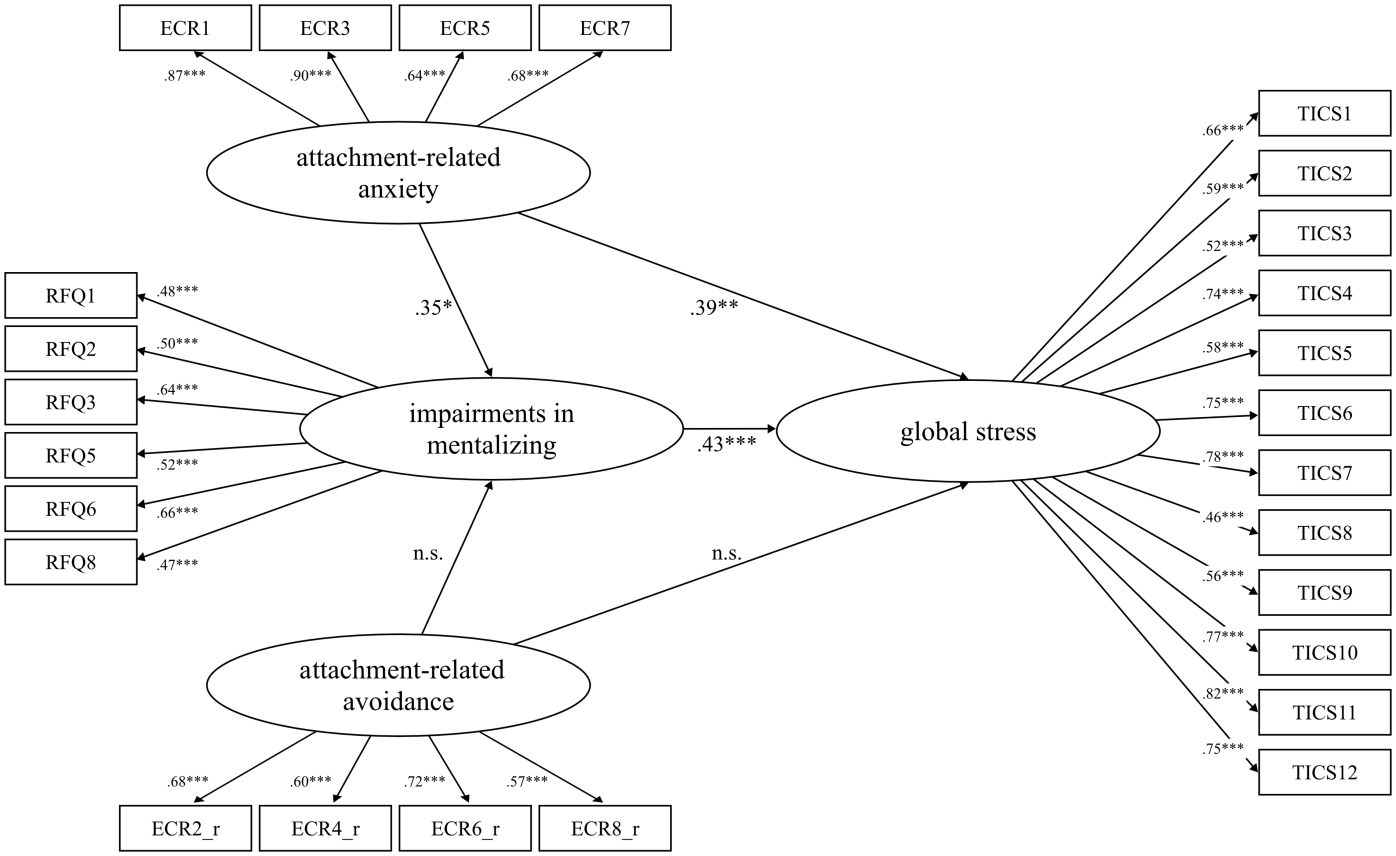
Structural Equation Model.

Figure 2 shows the model testing the main hypotheses. Data fit was acceptable (*χ*^2^(283, *n* = 202) = 420.23, *p* = 0.000, RMSEA = 0.05 [0.04, 0.06], CFI = 0.94, SRMR = 0.07). In the final model, covariates were excluded because age, native language and gender led to a decrease in model fit indices. Based on 10,000 bootstrap samples, positive associations of attachment-related anxiety on global stress experience (*β* = 0.39 [0.21–0.56], *p* = 0.001) and on impairments in mentalizing (*β* = 0.35 [0.11–0.53], *p* = 0.029) were found. Further, impairments in mentalizing were positively linked to the subjects’ global stress experience (*β* = 0.43 [0.26–0.60], *p* = 0.000). Attachment-related avoidance was neither associated with impairments in mentalizing (*β* = 0.24 [0.02 – –0.54], *p* = 0.079) nor with global stress experience (*β* = −0.18 [−0.34 – –0.03], *p* = 0.052). The association between attachment-related anxiety and stress experience was partially mediated by impairments in mentalizing (*β* = 0.15 [0.07–0.27], *p* = 0.016). In contrast, the indirect path of attachment-related avoidance on global stress experience through mentalizing was not significant (*β* = 0.10 [0.01–0.30], *p* = 0.069). In summary, direct and indirect effects exerted a total effect of *β* = 0.54 [0.39–0.68], *p* = 0.000) on global stress experience and accounted for a total of 39.8 percent of the variance in global stress experience.

## Discussion

The present study aimed to examine the associations between attachment insecurity and global stress experience in prospective teachers. Furthermore, it was tested whether this relationship was mediated by the individual’s mentalizing capacity.

Our results confirm Hypothesis 1, i.e., the expectation of a positive association between attachment-related anxiety and current stress experience in preservice teachers. Increasing attachment-related anxiety was linked to higher levels of stress experience which is consistent with the attachment theory’s framework. Specifically, due to early inconsistent caregiving experiences and a failure to co-regulate in stressful situations, individuals with insecure-anxious attachment styles develop internal working models in which they are represented as having low competence and self-efficacy in dealing with challenging situations (Ainsworth et al., 1978; Bretherton and Munholland, 2018). From this point of view, anxious attachment styles can be conceptualized as a hypervigilant state of mind due to the hardly predictable reactions of the attachment figure which systematically and negatively distorts the perception of mental states and capabilities – even many years later in adulthood (Ehrenthal et al., 2018; Tironi et al., 2021). As a result, anxious individuals have only few options available to cope with challenging, demanding, or threatening experiences, which rapidly evoke stress and elicit strong feelings of helplessness and loss of control (Dykas and Cassidy, 2011). This study was able to replicate this link from other studies (Fearon et al., 2010; Groh et al., 2012, 2017; Bordoagni et al., 2021; Brugnera et al., 2021) among prospective teachers. These findings are consistent with results suggesting that attachment-related anxiety represents a risk factor associated with psychosocial distress, mental health problems, and adverse development (Zhang et al., 2022).

Hypothesis 2 predicted a direct association between attachment-related avoidance and current stress experience in preservice teachers, but it was rejected in light of the result as there was no significant relationship between the two variables. This finding contradicted attachment theory’s notion that individuals with insecure-avoidant attachment representations experience high levels of stress more rapidly (Bowlby, 1988; Tironi et al., 2021) and the findings of Brugnera et al. (2021), who empirically confirm such a relationship among psychotherapists. However, due to the self-report instruments used in the current study, it is likely that in particular avoidant subjects responded in a way that could be interpreted as trivializing or suppressing potentially threatening self-states for the purpose of self-protection. This defense process could have contributed to the fact that threatening self-states or self-states such as current stress experiences were negated, thus influencing the response behavior. However, the result may also be explained by certain characteristics of the avoidant attachment pattern. It is possible that prospective teacher candidates with higher levels of attachment-related avoidance are more capable of distancing themselves emotionally from stress experiences, which is consistent with the attachment theoretical framework (Ainsworth et al., 1978). Children with avoidant attachment representations have learned to respond to high levels of stress by suppressing their emotions as a form of self-protection and coping mechanism (Dykas and Cassidy, 2011). The presented results suggest that many preservice teachers continue applying this coping mechanism when they experience high levels of stress in their professional development.

Hypothesis 3, which predicted a mediation of the direct association of both forms of attachment insecurity and global stress experience through mentalizing, can be partially confirmed. In the studied sample, a partial mediation effect was found for the association between attachment-related anxiety and stress experience, but not between attachment-related avoidance and stress experiences. Consequently, the effects documented by Brugnera et al. (2021) among psychotherapists can only be partially replicated among prospective teachers. The results suggest that attachment-related anxiety, predicted by own experiences of inconsistent and unreliable caregiving behavior by the attachment figure in childhood, promotes limitations in mentalizing capacity, which in turn is linked to an increase in general stress experiences. This finding is in line with the mentalizing framework (Fonagy et al., 2002), which postulates that the quality of early attachment experiences is a determinant factor in the development of effective mentalizing skills (Luyten et al., 2017). Furthermore, according to Fonagy et al. (2017), effective mentalizing enables the inner-psychic processing of aversive experiences, so that a coherent self-experience is maintained even under demanding circumstances.

In contrast, the lack of a significant indirect path from avoidant attachment representations to global stress experience through impairments in mentalizing in our sample suggests that early experiences of rejection in attachment relationships do not necessarily lead to compromised mentalizing. This contradicts the mentalization theory’s postulate that secure attachment representations in particular are linked to effective mentalizing (Fonagy et al., 1991, 2002). However, the mentalizing concept has increasingly focused on the extended social environment, emphasizing that the development of effective mentalizing is not only determined by early attachment experiences, but also by other social relationships (Fonagy and Allison, 2014), for example with peers, teachers, or spouses (Fonagy et al., 2017; Luyten et al., 2020). Therefore, this novel finding needs replication in future studies. However, individuals with avoidant attachment representations may tend to have a positively biased assessment of their own abilities for the purpose of self-protection, which is consistent with results from other studies showing less self-concept clarity in avoidantly attached subjects (e.g., Emery et al., 2018). Following attachment theory (Ainsworth et al., 1978), a positively biased self-perception in avoidant individuals is an adaptation to a non-responsive environment. It allows subjects to avoid any thoughts about oneself that are too difficult to acknowledge or recognize, because of the high degree of mental stress such a recognition would bring about.

### Limitations

Several limitations must be considered when interpreting the current results. First, causal inferences cannot be drawn due to the cross-sectional study design. Hence, all causal interpretations are based on theoretical assumptions that are merely consistent with the observations, making replications of the results in longitudinal research designs essential. Second, as only preservice student teachers were studied, no inferences can be made for the general teacher or in-service teacher population. Future studies need to replicate the results with (in-service) teacher samples. Third, the operationalization used in this study can bias the results. Only self-rating instruments were used, which are necessarily subjective. Similarly, the results may be affected by shared method variance, and the fact that mentalizing was exclusively assessed as uncertainty in the use of mental states, which is only one broad type of impairments in mentalizing (Fonagy et al., 2016). Future studies should make use of other forms of operationalization such as physiological measures, performance testing, or interviews in order to reduce potential bias. Fourth, future studies should specifically pay attention to the association between the avoidant attachment pattern, mentalizing impairments and global stress experiences, trying to replicate the finding.

### Implications

With reference to the high levels of stress experienced by teachers, it is unlikely that this can be fully explained by contextual factors that are typically associated with the profession of teaching, such as class size, school location, or characteristics of the students (Klusmann et al., 2008; Hillert et al., 2013). To a greater extent, variability in the levels of stress among teachers dependent on individual characteristics, and are rooted in ineffective coping strategies or a lack of social support (e.g., Schaarschmidt et al., 1999; Lehr et al., 2008). This emphasizes the importance of university training as a critical phase for professional development (Klusmann and Waschke, 2018), during which risk dispositions can be addressed proactively, i.e., before student teachers enter their profession. The results of this study suggest that raising awareness of student teachers’ insecure attachment and a lack of mentalization skills are risk factors for their own future stress levels as in-service teachers. As their own emotional well-being impacts the well-being and development of their students, targeted support for preservice teachers in addressing these challenges may be particularly important. Exploring one’s own attachment experiences can be beneficial, for example through mentalization-based supervision as it opens up the opportunity for preservice teachers to take a reflective approach to their own attachment histories and to raise their own awareness of the possible negative impact of insecure attachment on their professional development.

## Data availability statement

Data will be made available per request by the first author, N-HS, schwarzer@ph-heidelberg.de.

## Ethics statement

All subjects provided written informed consent to participate in the study. The research program was approved by the Ethics Committee of the Ludwigsburg University of Education (III-Sopead_NiSc-0009).

## Author contributions

N-HS: conception and design, execution, interpretation of the data and writing of the original draft. N-HS and LD: analysis. TN, PF, SG, TB, and LD: review and editing. All authors contributed to the article and approved the submitted version.

## Conflict of interest

The authors declare that the research was conducted in the absence of any commercial or financial relationships that could be construed as a potential conflict of interest.

## Publisher’s note

All claims expressed in this article are solely those of the authors and do not necessarily represent those of their affiliated organizations, or those of the publisher, the editors and the reviewers. Any product that may be evaluated in this article, or claim that may be made by its manufacturer, is not guaranteed or endorsed by the publisher.
